# Aesthetic appeal influences visual search performance

**DOI:** 10.3758/s13414-022-02567-3

**Published:** 2022-10-14

**Authors:** Irene Reppa, Siné McDougall

**Affiliations:** 1 School of Psychology, Swansea University, Swansea, SA2 8PP UK; 2 Department of Psychology, Bournemouth University, Bournemouth, UK

**Keywords:** Visual search, Aesthetic appeal, Visual complexity, Concreteness

## Abstract

Aesthetic appeal of a visual image can influence performance in time-critical tasks, even if it is irrelevant to the task. This series of experiments examined whether aesthetic appeal can act as an object attribute that guides visual search. If appeal enhances the salience of the *targets* pre-attentively, then appealing icons would lead to more efficient searches than unappealing targets and, conversely, appeal of *distractors* would reduce search efficiency. Three experiments (*N* = 112) examined how aesthetic appeal influences performance in a classic visual search task. In each experiment, participants completed 320 visual search trials, with icons varying in rated aesthetic appeal and either visual complexity (Experiments 1 and 2) of concreteness (Experiment 3) among two, four, eight, or 11 distractor icons. While *target* appeal did not influence search efficiency it sped up search times in all three experiments: appealing targets led to faster response time (RT) than unappealing targets across all experiments, and compared to neutral distractors, appealing *distractors* slowed search RT down. These findings are the first to show that an object’s aesthetic appeal influences visual search performance.

## Introduction

The term *aesthetic appeal*, or just *appeal*, refers to mild aesthetic experiences and is revealed by simple rating judgements made on the basis of liking (see Reber et al., 2004, for review). Aesthetics can influence our behaviour across a range of everyday life activities. We go to art galleries, fill our homes with things we like, and purchase products not only based on functionality but based on how much joy we get from interacting with them.

Aesthetic appeal influences not only our everyday behaviour but also our *performance* with the objects around us. This is not entirely surprising, because as a visual attribute of the world around us appeal is perceived extremely quickly – within 50 ms (e.g., Lindgaard et al., 2006) – rendering it a good candidate to influence time-critical performance. However, only a handful of studies using a variety of tasks have examined whether visual aesthetic appeal might influence performance and findings have been mixed (see Thielsch, Scharfen, et al., 2019b, for a recent review and meta-analysis). Some studies have found *no effect* of stimulus appeal on task performance (e.g., Hartmann et al., 2007; Sonderegger et al., 2012; Thüring & Mahlke, 2007; Tractinsky et al., 2000). Other studies have found positive effects of appeal on performance suggesting that appealing stimuli can *increase* performance efficiency (e.g., Moshagen et al., 2009; Reppa et al., 2021; Reppa & McDougall, 2015; Sonderegger & Sauer, 2010). In contrast, *decreased* performance efficiency for appealing stimuli has sometimes been reported (e.g., Ben-Bassat et al., 2006; Meyer et al., 1997; Sauer & Sonderegger, 2009, 2011; Tufte, 1983).

The current study used a classic visual search task to determine whether appeal is a visual attribute that can guide the deployment of attention: of particular interest was whether any performance boost due to appeal affected *search efficiency* or *search times*. Use of classic visual search tasks allow calculation of search slopes based on the linear relationship between search time and set size. Search slopes are commonly used to determine whether stimulus dimensions – such as aesthetic appeal – can be attributes that efficiently guide the deployment of attention (e.g., see Wolfe & Horowitz, 2004, 2017, for reviews). In general, a stimulus attribute can be classed as attention-guiding, when it is independent of set size and its search slope is flat at near zero. Such flat slopes are typically called very efficient, while slopes of between 5 and 10 ms are called quite efficient, and slopes of over 10 ms inefficient.

Research to date suggests that stimulus attributes rarely produce near zero search slopes although attributes that are threatening or evolutionarily relevant are notable exceptions (Becker et al., 2011; Eastwood et al., 2001; Fox et al., 2001; Öhman et al., 2001). Many stimulus dimensions, however, lead to more or less efficient searches without necessarily producing ‘pop-out’ effects (Golan et al., 2014; Hershler & Hochstein, 2005; see also Frischen et al., 2008 for a review). Alternatively, a stimulus attribute may simply speed up or slow down search times without affecting search efficiency, i.e., there is no effect on the search slope but overall response times (RTs) during search are changed (Della Libera & Chelazzi, 2009; Lee & Shomstein, 2014).

In the present investigation we examined the effect of aesthetic appeal on visual search performance. However, stimulus characteristics known to influence performance are highly correlated with appeal and are known to have a significant effect on performance. Perceptions of aesthetic appeal are strongly influenced by several stimulus dimensions (e.g., colour: Bonnardel et al., 2011; Palmer et al., 2013; concreteness: Kawabata & Zeki, 2004; familiarity: Reber et al., 2004; Reppa & McDougall, 2015; symmetry and harmony: Palmer & Griscom, 2013; visual complexity: Eisenman, 1967; Reppa et al., 2021). Similar stimulus dimensions including visual complexity, concreteness, and familiarity are also known to influence performance in search and localisation tasks (e.g., Byrne, 1993; Isherwood et al., 2007; Jacobsen & Höfel, 2002; Kawabata & Zeki, 2004; McDougall et al., 2000; McDougall & Reppa, 2008; Vartanian & Goel, 2004).

In order to conduct this research in a well-controlled manner, we needed a micro-world of well-defined and controlled stimuli that allow examination of the aesthetic appeal-performance relationship, while carefully controlling for confounding variables. Icons are such a micro-world, not least because their characteristics are well documented both regarding their relationship with ratings of appeal and regarding task performance (e.g., McDougall et al., 1999; McDougall et al., 2000; McDougall & Reppa, 2008). McDougall and Reppa (2008) found that *three* icon characteristics in particular, familiarity, concreteness, and visual complexity, accounted for a significant amount of the variance in aesthetic appeal ratings.

In sum, visual complexity, concreteness, and familiarity contribute to (e.g., Jacobsen & Höfel, 2002; Kawabata & Zeki, 2004; Martindale et al., 1988; Vartanian & Goel, 2004; Zajonc, 1968, 2000), while also being strongly correlated with (e.g., McDougall & Reppa, 2008), ratings of aesthetic appeal while at the same time having been shown to affect performance (e.g., Byrne, 1993; Green & Barnard, 1990; Isherwood et al., 2007; McDougall et al., 2000; McDougall et al., 2006; McDougall & Isherwood, 2009; Rogers & Oborne, 1987; Scott, 1993; Stotts, 1998). Therefore, although icons (especially those used in the current investigation) may not elicit the kind of strong emotive response one may associate with a strong aesthetic experience, they are known to elicit reliable appeal and emotional responses (e.g., McDougall & Reppa, 2008; Prada et al., 2016). Moreover, they offer an ideal stimulus set to systematically examine the effect of aesthetics on performance, allowing control of stimulus factors, to ensure that any effects of appeal do not actually reflect effects of confounding factors contributing to appeal and performance.

In Experiments 1 and 2 icon appeal and visual complexity were varied orthogonally while holding icon concreteness and familiarity constant. This made it possible to examine the relative effects of appeal and complexity on search without being affected by other icon attributes likely to affect search. In Experiment 1 we examined the effect of appeal and visual complexity on search times and search slopes to examine if appeal has an independent effect on the speed of search (search RT) and on search efficiency (search RT slopes). Appeal was defined both by using pre-existing normative appeal ratings for the icons employed in the task (McDougall & Reppa, 2008) as well as individual participants’ own subjective ratings of appeal. This allowed us to examine whether independently and subjectively defined appeal yields similar influence on search performance. Experiment 2 extended Experiment 1 to examine the effect of appealing distractors on search performance. Experiment 3 focused on icon appeal and concreteness, and appeal and familiarity varying these icon attributes orthogonally, while holding icon complexity constant.

Previous work has suggested that appealing stimuli may be inherently rewarding (e.g., Kirk et al., 2009; Reppa et al., 2021). Furthermore, there is evidence to show increased efficiency for stimuli we personally like and thus find more rewarding (e.g., Della Libera & Chelazzi, 2009; Lee & Shomstein, 2014). Therefore, *appeal may increase motivation*, which in turn can speed up search performance and increase performance efficiency, especially for those icons that are subjectively appealing. If appealing stimuli act as other rewarding stimuli, like food or money, then they may be processed faster than their unappealing counterparts.

## Experiment 1: The effect of target complexity and target appeal on visual search

Experiment 1 examined the effect of aesthetic appeal of target icons on visual search performance. Distractors were chosen that were rated as neutral in terms of appeal (see Table 1). Targets varied orthogonally in terms of previously rated aesthetic appeal (based on the ratings obtained in McDougall & Reppa, 2008) as well as rated visual complexity, while keeping concreteness and familiarity constant (based on ratings obtained in McDougall et al., 1999). Based on previous evidence (e.g., Krasich et al., 2019; Reppa & McDougall, 2015; Sonderegger & Sauer, 2009) it was hypothesized that aesthetically appealing icons would have significant influence on task performance, reducing response times, compared to unappealing ones. Importantly, the use of a classic visual search task made it possible to examine whether appeal of a target stimulus is pre-attentively processed and guides attention more efficiently around the display. If appeal acts as an attention-guiding attribute, then appealing targets would lead to more efficient searches – the search slopes for appealing targets would be shallower than the slopes for unappealing targets.

**Table 1:** Materials employed in Experiment 1 and Experiments 2a and 2b

(A) Mean Likert-scale ratings (and standard deviations) for Targets and Neutral Distractors (used in Experiments 1 and 2a) and Appealing Distractors (used in Experiment 2b)						
Icon characteristics	Appealing Complex **AC** (N=10)	Appealing Simple **AS** (N=10)	Unappealing Complex **UC** (N=10)	Unappealing Simple **US** (N=10)	Neutral Distractors **ND** (N=72)	Appealing Distractors **AD** (N=72)
Appeal	3.50 (0.88)	3.50 (0.53)	2.45 (0.15)	2.61 (0.10)	3.00 (0.40)	3.45 (0.27)
Complexity	3.49 (0.15)	1.68 (0.80)	3.69 (0.26)	1.82 (0.23)	2.55 (0.77)	2.46 (0.73)
Concreteness	3.85 (1.11)	3.61 (0.88)	3.26 (0.90)	2.27 (0.84)	3.18 (0.89)	3.58 (0.96)
Familiarity	3.19 (0.62)	3.59 (0.94)	2.70 (0.87)	2.96 (0.85)	2.87 (0.90)	3.26 (0.87)
(B) Results of one-way analyses of variance and Newman-Keuls Comparisons examining differences between 1-5 Likert scale ratings for (a) target icon types (b) target icons and neutral distractors (c) target icons and appealing distractors. The symbols ‘>’ and ‘<’ mean higher and lower ratings respectively, while the ‘=’ symbol means no difference in the rated dimension						
	(a) Comparisons between target icon types		(b) Comparisons between target icon types and neutral distractors		(c) Comparisons between target icon types and appealing distractors	
Icon characteristics	ANOVA result	Newman-Keuls comparisons of each icon type	ANOVA result	Newman-Keuls comparisons of icon types with distractors	ANOVA result	Newman-Keuls comparisons of icon types with distractors
Appeal	F(3,36)=40.03, p<.001	AC=AS>UC=US	F(4,107)=18.83, p<.001	AC=AS>ND UC=US<ND	F(4,107)=45.30, p<.001	AC=AS=AD UC=US<AD
Complexity	F(3,36)=48.48, p<.001	AC=UC>AS=US	F(4, 107)=19.54, p<.001	AC=UC>ND AS=US<ND	F(4,107)=22.68, p<.001	AC=UC>AD AS=US<AD
Concreteness	F<1, p>.05 p<.001	AC=AS=UC=US	F(4, 107)=1.26, p=.29	AC=AS=UC=US=ND	F(4,107)=0.76, p=.55	AC=AS=UC=US=AD
Familiarity	F(3,36)=2.17, p>.05	AC=AS=UC=US	F(4, 107)=1.89, p=.12	AC=AS=UC=US=ND	F(4,107)=2.18, p=.08	AC=AS=UC=US=AD

Prior to the visual search task, participants provided ratings of appeal for all the target icons that were later used as targets in a visual search task. Obtaining appeal ratings from participants of the target icons allowed us to examine whether any effect of appeal on performance would be stronger for targets defined by appeal ratings obtained from the participants themselves rather than ratings provided prior to the experiment by others, as is normally the case.

Previous research on attentional capture – a task where the nature of distractors is manipulated to examine its effect on performance with target stimuli – has shown that attention can be captured by distractors that are appealing to the participants themselves (e.g., favourite team logos), but not by distractors that are neutral to the participant (other team logos; e.g., Biggs et al., 2012; Krasich et al., 2019). Therefore, it may be the case that any effect of appeal on performance is stronger when participants are looking for targets that they themselves have rated as appealing, compared to those that they rated as neutral or unappealing.

### Method

#### Participants

Forty undergraduate and postgraduate Swansea University students, 34 females and eight males, took part in the study in exchange for participant pool credits. The usual sample size for visual search experiments manipulating variables within-participants is between ten and 25 participants. For current study G*Power (3.1) analysis suggested a minimum of 18 to obtain a medium effect size (.06) for the key Appeal and Set Size interaction. Here we recruited a little over double the required participants ensuring a sufficient number of subjective icon ratings. Specifically, as participants would be asked to rate icons in terms of attractiveness, we needed to avoid missing values for some categories for some participants. Participant ages ranged from 19 to 35 years (*M* = 22.95, *SD* = 3.81). Recruitment primarily took place via the Psychology Department’s participant pool, with additional students sourced via posters across the University’s campus. The study was approved by the College of Human & Health Sciences, Swansea University, Research Ethics Committee.

#### Apparatus and materials

In the search task, 40 target icons and 72 distractor icons were chosen from the icon corpus in McDougall et al. (1999). Stimuli were all black and appeared against white background. They were viewed from approximately 60 cm (no headrest was used but we ensured participants were sitting so that their eyes were at a distance of 60 from the monitor). From that distance, all icons measured approximately 2^o^ × 2^o^ of visual angle. The placeholders the icons appeared within, were squares measuring 4^o^ × 4^o^ with a border of 3 pixels, and a distance of 2^o^ between them.

**Fig. 1:**
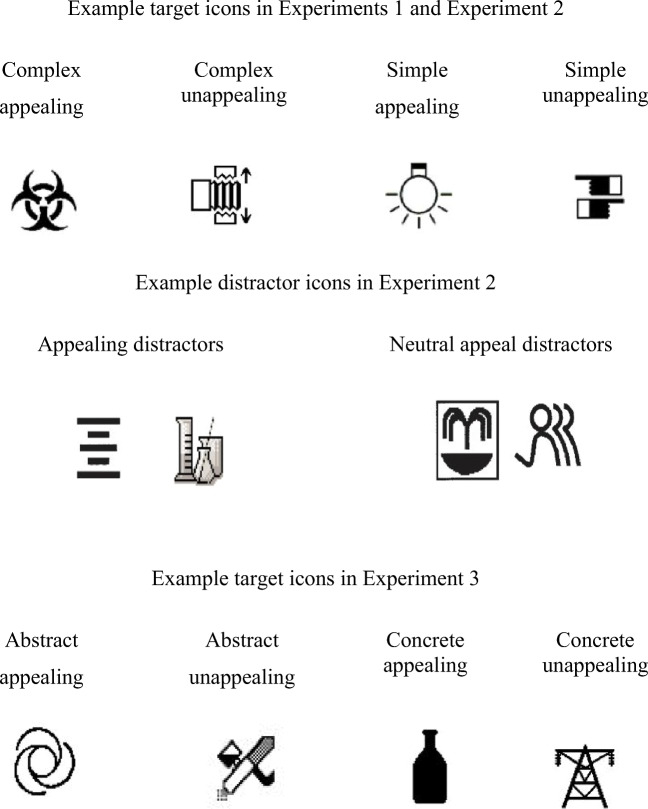
Examples of target and distractor icons used in the current experiments

The ratings of visual complexity and visual aesthetic appeal were used to vary the target icons orthogonally. Four icon types were created: appealing complex, appealing simple, unappealing complex, and unappealing simple (Fig. 1). Ratings of visual complexity, concreteness, and familiarity were obtained from McDougall et al. (1999) and ratings of appeal were obtained by McDougall and Reppa (2008). Table 1 shows the findings from univariate ANOVAs examining differences between icon types: icons differed significantly in terms of their rated visual complexity and appeal but did not differ in terms of concreteness and familiarity.

Seventy-two icons from the original icon corpus were used as distractors, whose characteristics (visual complexity, concreteness, familiarity, and appeal) appear in Table 1a. Distractors were chosen to be neutral in terms of rated appeal. Comparisons between target and distractor icons in terms of appeal, visual complexity, concreteness, and familiarity appear in Table 1b. To obtain individual, rather than normative, ratings participants were asked to rate 60 icons (40 icons that were targets in the visual search task and 20 filler icons). The 20 filler icons were from the original icon set in McDougall et al. (1999) and were chosen so that they were neutral in terms of appeal (based on ratings obtained by McDougall & Reppa, 2008). Table 2 shows the appeal ratings given by the participants to the icons previously identified as appealing, unappealing and neutral (here used as filler trials in the rating task) and based on McDougall and Reppa’s (2008) findings.

**Table 2:** Experiment 1: Previously obtained mean normative ratings (and standard deviations) and participants’ own ratings of appealing, unappealing and neutral filler icons

	Normative ratings McDougall and Reppa (2008)	Participants’ own ratings
Appealing	3.50 (0.37)	3.41 (1.12)
Unappealing	2.53 (0.15)	2.54 (0.97)
Neutral filler items	3.18 (0.03)	3.20 (1.04)

*Note.* The filler icons were only included in the ratings booklet and were selected to be of medium levels of appeal

#### Design

The visual search task was based on a 2 (Target Presence: present vs. absent) × 4 (Set Size: 3, 6, 9, 12) × 2 (Complexity: complex vs. simple) × 2 (Appeal: appealing vs. unappealing) repeated-measures design yielding 32 experimental conditions. There were ten trials per condition trials per condition, yielding 320 trials per participant. The dependent measure was RT.

#### Procedure

Participants were tested individually in a quiet well-lit laboratory. Participants first completed the rating task followed by a computer-based visual search task. For the rating task, each participant was presented with a booklet of 60 icons which included instructions and examples. Participants rated each icon by circling a Likert scale response from 1 (Really Dislike) to 5 (Really Like). The booklets started on different pages for each participant to avoid any potential order effects.

**Fig. 2:**
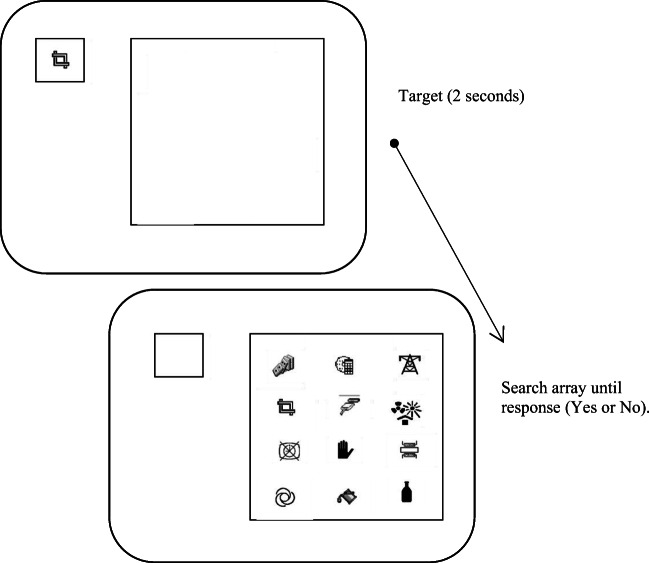
Example of a target present experimental trial with a search array of 12 items. The target appeared alone on the top left of the screen for 2 s. After 2 s, the target disappeared and the array of icons (3, 6, 9 or 12) appeared until response

Following the ratings task participants carried out the visual search task. Trial presentation and recording of responses was controlled via PsyScope (Cohen et al., 1993) run on a Mac OS11, connected to a 19-in. Samsung flat-screen monitor. An example trial is illustrated in Fig. 2. In each trial, the target icon was presented in the top left of the screen for 2 s. Following target offset, an array of three, six, nine or 12 icons appeared on the right part of the screen within a 3 × 4 grid. The array remained visible until the participant responded. Half of the participants indicated whether the target was present among those icons by pressing the key ‘M’ or absent by pressing the key ‘Z’. The assignment of keys was reversed for the remaining half of the participants. Participants were prompted to respond as fast and accurately as possible, and told that the target would be present 50% of the time. Incorrect responses (e.g., stating that a target icon was present in the array when it was not, and vice versa) were followed by a 500-ms beep sound. Target and distractor icons were presented randomly in each of the 12 possible locations. Presentation of each of the 32 conditions was fully randomised across a 45-min experiment with three chances to take a break at approximately equal intervals. Each icon was presented once as a target, and eight times as a distractor. None of the 20 filler icons from the participants’ rating booklet appeared in the visual search task.

#### Data analysis

For all experiments reported here, separate analyses were carried out on target-present and target-absent search data. The dependent measures examined were correct mean RT in milliseconds, and search slopes. In all experiments, RT that was 3 standard deviations above or below the mean per participant per condition was removed from the data and not analysed further. Those removals accounted for no more than 1.6% of the data in any of the experiments. Search slopes of correct RT × Set Size are the most commonly used index of the efficiency of the search. Search slopes give an estimate of the cost of adding an item to the visual display (Wolfe, 2001). Partial eta-squared (*ηp*^*2*^) was reported for all significant effects (Cohen, 1973), to indicate the proportion of the variance in response times and slopes attributable to each variable separately. Finally, Cohen’s *d* was reported for all pairwise comparisons investigating significant interactions.

### Results

In the rating task, the majority (38 out of the 40) of participants used the full scale of appeal ratings. No participants used fewer than 3 points on the Likert scale. Appeal ratings per icon type are shown in Table 2. Overall, icons deemed appealing using the McDougall and Reppa (2008) norms were more likely to be rated as appealing than neutral or unappealing and, similarly, unappealing icons were more likely to be rated as unappealing than appealing or neutral.

For the visual search task, errors (4.29%) were removed from the data and analysed separately. Correct responses that were ±3 SDs from the mean per participant per condition (1.5% of correct trials) were classed as outliers, removed from the data, and not analysed further.

### RT analyses using independent appeal ratings

**Fig. 3:**
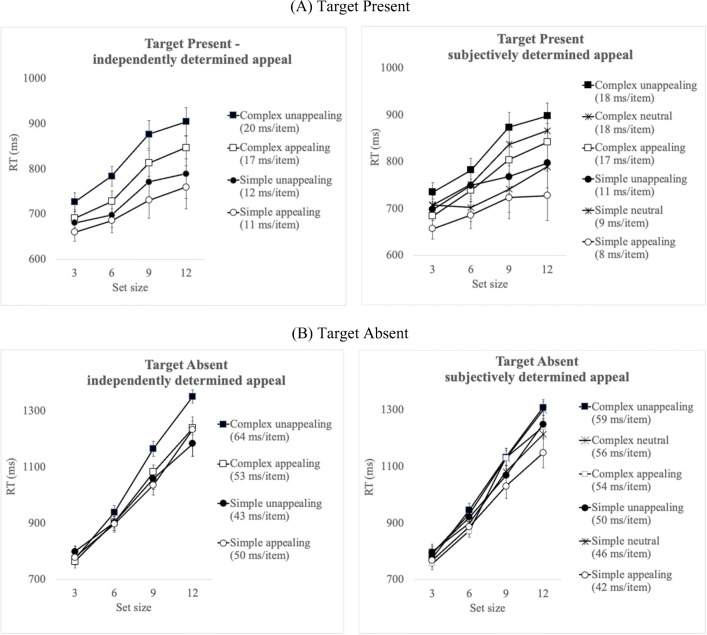
Mean correct response time (RT) per condition for target-present trials (**a**) and target-absent (**b**) trials in Experiment 1. Means appear separately for target icons that were independently or subjectively appealing (see text for details). The search slope for each icon type appears in parentheses

In this set of analyses, appealing and unappealing icons were determined based on ratings provided by participants in McDougall and Reppa (2008). Separate analyses were carried out for target present and target-absent RT. Correct mean RT per condition is shown in Fig. 3. Table 3 presents a summary of the findings from the statistical analyses reported below.

**Table 3:** Experiment 1: Summary of findings from analyses with appeal scores based on McDougall and Reppa’s (2008) ratings and on participants’ own ratings. Effect sizes are only provided for significant effects

	McDougall and Reppa (2008) normative ratings of appeal				Participants’ own ratings of appeal			
Target present	*df*	*F*	*p*	*ηp*^*2*^	*df*	*F*	*p*	*ηp*^*2*^
RT analyses								
Set size	3,117	82.96	<.001	.68	3,96	36.16	<.001	.53
Complexity	1,39	119.26	<.001	.75	1,32	34.28	<.001	.52
Appeal	1,39	40.34	<.001	.51	2,64	13.33	<.001	.29
Complexity × Appeal	1,39	4.75	.03	.11	2,64	0.55	.58	-
Set Size × Complexity	3,117	5.38	.003	.12	3,96	6.82	<.001	.18
Set Size × Appeal	3,117	0.63	.59	-	3,96	0.50	.81	-
Set Size × Complexity × Appeal	3,117	0.31	.81	-	6,192	0.26	.95	-
Slopes analyses								
Complexity	1,39	15.49	<.001	.28	1,39	27.23	<.001	.41
Appeal	1,39	1.79	.19	-	1,39	0.06	.94	-
Complexity × Appeal	1,39	0.09	.76	-	1,39	0.31	.74	-
Target absent	*df*	*F*	*p*	*ηp*^*2*^	*df*	*F*	*p*	*ηp*^*2*^
RT analyses								
Set size	3,117	152.50	<.001	.80	3,99	125.91	<.001	.79
Complexity	1,39	21.79	<.001	.36	1,33	10.41	<.001	.24
Appeal	1,39	10.01	.003	.20	2,66	9.48	<.01	.23
Complexity × Appeal	1,39	8.97	.005	.19	2,66	0.02	.98	-
Set Size × Complexity	3,117	7.75	.001	.17	3,99	4.72	<.01	.12
Set Size × Appeal	3,117	1.46	.34	-	6,198	1.07	.38	-
Set Size × Complexity × Appeal	3,117	3.06	.03	.07	6,198	0.85	.53	-
Slopes analyses								
Complexity	1,39	13.84	<.001	.26	1,39	9.39	.004	.19
Appeal	1,39	2.53	.12	-	2,78	0.94	.39	-
Complexity × Appeal	1,39	6.32	.02	.14	2,78	2.14	.15	-

#### Target-present RT

A 4 (Set Size: 3, 6, 9, 12) × 2 (Complexity: complex versus simple) × 2 (Appeal: appealing vs. unappealing) repeated-measures ANOVA, with appeal determined based on the ratings obtained by McDougall and Reppa (2008), was carried out on correct RT.

The main effect of Set Size was significant with RT increasing as set size increased. The main effects of Complexity and of Appeal were also significant. There was also a significant Complexity × Appeal interaction: pairwise comparisons confirmed that search RT was faster for complex appealing compared to complex unappealing icons, *t*(39) = 5.10, *p* < .001, *d* = .81. The same difference was observed between simple appealing and unappealing icons, but the effect size was smaller, *t*(39) = 3.87, *p* = .003, *d* = .61. The Set Size × Complexity interaction was also significant with shallower slopes for simple compared to complex icons (see target-present slopes analysis below). There were no other significant interactions.

#### Target-present slopes

The RT by set size slopes for *target-present* trials were submitted to a 2 (Complexity: complex vs. simple) × (Appeal: appealing vs. unappealing) repeated-measures ANOVA. There was only a significant main effect of Complexity with steeper slopes for complex (18 ms/item) compared to simple icons (14 ms/item). The main effect of Appeal was not significant and neither was the Complexity × Appeal interaction.

Overall, icon *appeal* interacted with *visual complexity* to boost search times when the target was present in the array – *search RT* was faster when targets were appealing compared to unappealing icons with this difference being larger for unappealing complex icons. In contrast, *search efficiency* was influenced by complexity only, with shallower slopes for visually simple compared to visually complex icons.

#### Target-absent RT

A repeated-measures ANOVA for *target-absent* RT showed a significant main effect of Set Size with increasing RT as the number of distractors increased. The main effects of Complexity and Appeal were significant as was Complexity × Appeal interaction. Pairwise comparisons to examine the interaction, showed that target-absent RT was overall faster when looking for complex appealing compared to unappealing targets, *t*(39) = 4.73, *p* < .001, *d* = .75, while there was no difference in search RT between simple appealing and simple unappealing target, *t*(39) = .29, *p* = .78, *d* = .05.

There was also a significant interaction between Set Size and Complexity, *F*(3, 117) = 7.75, *p* < .001, *ηp*^*2*^ = .17, reflecting steeper RT slopes for complex compared to simple targets (see target-absent slopes analyses below). The Set Size × Appeal interaction was not significant *but the significant three-way interaction was significant*. The three-way interaction was driven by the interaction between Complexity and Appeal on search slopes (see slopes analysis below).

#### Target-absent slopes

Search slopes for *target-absent* trials were submitted to a 2 (Complexity: complex vs. simple) × (Appeal: appealing vs. unappealing) ANOVA and reveal a significant main effect of Complexity but not Appeal. The Complexity × Appeal interaction was significant: pairwise comparisons confirmed that search slopes when looking for complex targets were shallower when they were appealing (53 ms/item) compared to when they were unappealing (64 ms/item), *t*(39) = 2.60, *p* < .001, *d* = .41, with no significant difference between simple appealing and simple unappealing slopes (50 and 43 ms/item respectively), *t*(39) = 1.95, *p* = .07, *d* = .25.

Overall, for target-absent trials *appeal* interacted with *visual complexity* to influence overall *search RT*: search was terminated later for unappealing compared to appealing targets, with this difference being larger for complex compared to simple target icons. *Search efficiency* was also determined by the interaction between visual complexity and appeal, with shallower search slopes when searching for appealing targets, especially when they were complex.

### RT analyses using participants’ own appeal ratings

Appeal was coded separately for each participant depending on their own ratings of the target icons. That means that an icon rated as high in appeal for one participant may have been rated as neutral or unappealing by another. As before separate analyses were carried out for target-present and target-absent trials. The results of these analyses are shown in Table 3.

#### Target-present RT

A 4 (Set Size: 3, 6, 9, 12) × 2 (Complexity: complex versus simple) × 3 (Appeal: appealing, neutral, unappealing) repeated-measures ANOVA, with appeal determined based on each participant’s subjective appeal ratings, was carried out on correct RT. The main effect of Set Size was significant with reaction times increasing as set size increased. The main effects of Complexity and Appeal were significant but, this time, the Complexity × Appeal interaction was not significant. The Set Size × Complexity interaction was significant revealing shallower slopes for simple compared to complex icons (see target-present slopes analysis below). There were no other significant interactions.

#### Target-present slopes

The RT by set size slopes for *target-present* trials were submitted to a 2 (Complexity: complex vs. simple) × (Appeal: appealing vs. unappealing) repeated-measures ANOVA. There was only a significant main effect of Complexity with steeper slopes for complex (18 ms/item) compared to simple icons (9 ms/item). The main effect of Appeal was not significant and neither was the Complexity × Appeal interaction.

Overall, both *icon appeal* and *visual complexity* independently boosted search times when the target was present in the array – *search RT* was faster when targets were appealing compared to when unappealing, and faster when they were simple compared to visually complex. In contrast to the pattern of results when appeal was based on pre-existing ratings, when appeal was determined subjectively, there was no longer an interaction between appeal and complexity.

*Search efficiency* was only influenced by complexity, with shallower slopes for visually simple compared to visually complex icons. Nevertheless, the slopes for simple appealing and simple neutral icons were less than 10 ms per item which is in the ‘quite efficient’ search range.

#### Target-absent RT

A repeated-measures ANOVA for *target-absent* RT showed a significant main effect of Set Size, with increasing RT as the number of distractors increased, as well as Complexity and Appeal. The Complexity × Appeal interaction was not significant. There was a significant interaction between Set Size and Complexity reflecting steeper RT slopes for complex compared to simple targets (see target-absent slopes analyses below). No other interactions were significant.

#### Target-absent slopes

Search slopes for *target-absent* trials were submitted to a 2 (Complexity: complex vs. simple) × (Appeal: appealing vs. unappealing) ANOVA, showed only a significant main effect of Complexity with steeper slopes when looking for complex (56 ms/item) compared to simple (46 ms/item) targets. The main effect of Appeal was not significant and neither was Complexity × Appeal interaction.

Overall, for target-absent trials both *appeal* and *visual complexity* influenced overall *search RT*: search was terminated later for unappealing compared to appealing targets, and for complex compared to simple targets. *Search efficiency* was only determined by visual complexity, with steeper search slopes when looking for complex targets.

### Discussion

Experiment 1 examined whether appeal may be an attention-guiding visual object attribute. Participants searched displays of icons for targets that were orthogonally manipulated along appeal and visual complexity. The key findings were as follows. First, both visual complexity and appeal of target icons influenced search times, with faster search times for simple as opposed to complex icons, and for appealing compared to unappealing icons. However, only visual complexity influenced search efficiency. Second, appeal facilitated search regardless of whether it was independently or subjectively defined (i.e., whether what was considered appealing was determined by previous normative ratings or by the ratings provided individually by current participants). Third, appeal interacted with visual complexity when appeal was defined independently (based on previous ratings) – but not when appeal was defined subjectively (based on each participant’s ratings in the current study). Those findings are discussed in turn.

### Search efficiency

Visual complexity significantly slowed down search times and led to more inefficient searches. Previous work using search tasks without manipulating search array size has found a negative impact of visual complexity icons and symbols on performance (e.g., Byrne, 1993; Gerlach & Marques, 2014; Isherwood et al., 2007; McDougall et al., 2000; McDougall, Scott, 1993; Reppa et al., 2008; Reppa & McDougall, 2015; McDougall et al., 2006). The only other study that has to our knowledge examined the effect of visual complexity on a search task was carried out by Sun and Firestone (2021). In a series of experiments, they manipulated the visual complexity of target and distractor geometric shapes. Their findings were complimentary to those found here: when visually complex targets were embedded in a set of simple distractors, visually simple distractors led to easier processing and faster rejection (see also Wolfe & Horowitz, 2017, for a similar discussion and McDougall et al., 2000, for discussion of distinctiveness effects when target icons are embedded in contrasting distractors).

### Search speed

Visual search performance was not blind to appeal in the current study. Target appeal boosted search performance corroborating previous evidence showing that appealing icons are localised faster than unappealing icons (e.g., Reppa & McDougall, 2015; Reppa et al., 2021), and improved search times and memory for preferred websites (e.g., Baughan et al., 2020). However, a key question that a classic visual search task can address is whether or not a particular object feature or attribute can guide attention during a search task. As noted earlier, efficient searches are typically less than 10 ms per item and are thought to reflect pre-attentive processing of that feature while inefficient searches are typically greater than 10 ms per item and suggest that the target feature is not pre-attentively processed and not used to guide attention across the display. Even when appeal was determined based on participants’ own ratings, there was no difference in search slopes between appealing and unappealing targets. Therefore, although aesthetic appeal can influence visual search performance overall, there was no evidence in Experiment 1 that it is registered pre-attentively and guides visual search.

The faster search times for appealing targets suggests that appeal may lead to better processing, particularly since search for unappealing targets was longer for both target-present and target-absent trials. The longer search times for unappealing targets suggests that unappealing targets are processed less effectively than appealing targets, corroborating previous evidence using different tasks (e.g., Reppa & McDougall, 2015; Thielsch, Haines, & Flacke, 2019a).

### ‘Independent’ versus ‘subjective’ appeal

Finally, Experiment 1 revealed a difference in the effect of appeal on search depending on whether appeal was determined based on previous normative ratings (‘independent’ appeal) or based on the ratings of the participant themselves (‘subjective’ appeal). Specifically, when appeal was defined independently, based on previous ratings, it interacted with visual complexity: appeal sped up search times but only for complex icons, while search times were not affected by target appeal when icons were simple. This pattern of results replicates previous findings with icons and websites showing that appeal can influence performance but only under task duress (i.e., when looking for a visually complex target or through hard to navigate websites; Moshagen et al., 2009; Reppa & McDougall, 2015). However, a more ubiquitous effect of appeal was revealed when appeal was defined subjectively – target icons that the participants themselves found appealing (based on their own ratings), were found faster regardless of whether they were visually complex or simple. One reason for the ubiquitous effect of appeal on performance when appeal was determined subjectively may be that appealing icons were *only* those rated highly in terms of appeal, and unappealing icons were *only* those rated very low in appeal. All other icons were categorised as neutral. Search performance closely followed those categorisations – appealing icons were found faster than unappealing icons and neutral icons were in-between. Therefore, subjective ratings of appeal allowed us to observe the effect of highly appealing compared to highly unappealing stimuli on search performance. Under those circumstances, the effect of appeal was not conditional on visual complexity but had a strong independent influence on performance.

## Experiment 2: The effects of neutral versus appealing distractors on visual search

The purpose of Experiment 2 was twofold. First, to replicate the effects observed in Experiment 1 and second to examine the effect of target-distractor similarity in terms of appeal on search performance. Participants searched for appealing and unappealing targets among *neutral* distractors in Experiment 2a and among *appealing* distractors in Experiment 2b.

According to a saliency account, target-distractor similarity should uniquely determine search efficiency, with more efficient searches when the target defined by a specific attribute or feature differs from the average distribution of the distractors on that feature (e.g., Itti & Koch, 2000; Rosenholtz, 1999; Treisman & Gelade, 1980). So, when distractors are appealing, unappealing targets should be more salient, resulting in better visual search performance and more efficient search slopes. However, appealing targets should be less salient amongst the appealing distractors, resulting in poorer visual search performance.

Alternatively, search can be determined by whether the target contains the attribute in question, as opposed to the overall target-distractor similarity on that attribute. If that is the case, then unappealing targets lack the attribute of appeal which is present in appealing distractors. When searching for a target that is defined by the absence of feature – e.g., searching for an O among Qs, then search slopes are steeper than when searching for a target that is defined by a feature that is present, e.g., searching for a Q among Os (e.g., Wolfe, 2001). Therefore, if aesthetic appeal is a unique visual attribute determining search performance, then when distractors are appealing, unappealing targets should not guide search as they lack the attribute of appeal – search slopes for unappealing target should be no different between Experiments 2a and 2b. Meanwhile, search for appealing targets should be more inefficient when surrounded by appealing distractors compared to neutral distractors – thus, search slopes for appealing targets should be steeper in Experiment 2b (appealing distractors) compared to Experiment 2a (neutral distractors).

Previous work has shown similarly that high-reward distractors can capture attention and thus slow search performance (e.g., Anderson et al., 2011). If appealing stimuli act as rewarding stimuli do, then it may be harder to disengage from them. Therefore, we would expect steeper search slopes when distractors were appealing (Exp. 2b) compared to neutral (Exp. 2a).

## Experiment 2a: Neutral distractors

### Method

#### Participants

Twenty-six university-age undergraduate students (18 females and eight males) were recruited in exchange of participant pool credits. Participants had normal or corrected-to-normal vision and were naïve to the aim of the experiment. The study was approved by the College of Human & Health Sciences, Swansea University, Research Ethics Committee.

#### Apparatus and materials

The same apparatus was used as in Experiment 1. The target and distractor icons were identical to those used in Experiment 1. Distractor icons were all neutral in terms of aesthetic appeal and visual complexity (see Table 1). Appeal was defined based on the ratings obtained by McDougall and Reppa (2008).

#### Design, procedure and analyses

The design and procedure were identical to that of Experiment 1. The statistical analyses were identical to those employed in Experiment 1.

### Results

**Fig. 4:**
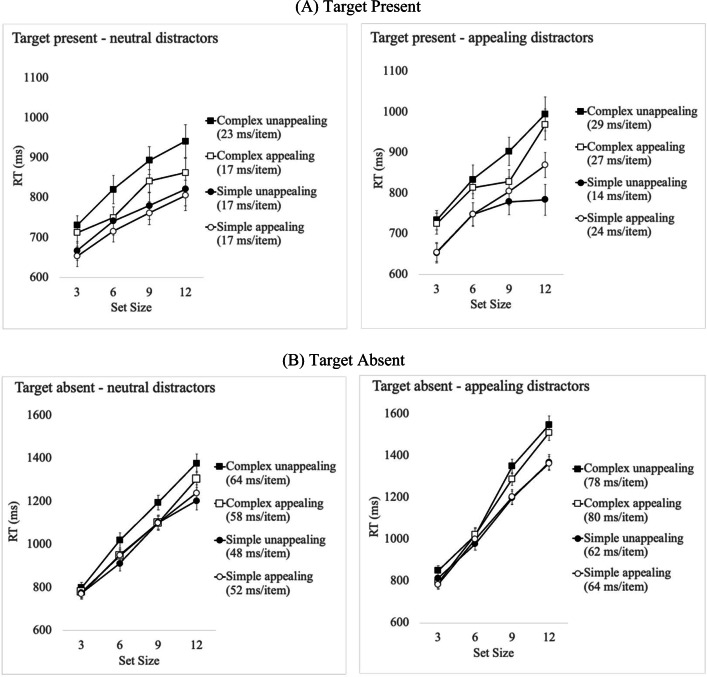
Mean correct response time (RT) per condition for target-present (a) and target-absent (b) trials, in Experiment 2a where distractors were of neutral rated appeal, and Experiment 2b where distractors were appealing. Error bars indicate standard error of the mean

Error trials accounted for 5.50% of all trials with 1.2% false alarms (saying yes to a target-absent trial) and 4.2% misses (saying No in a target-present trial). All error trials were removed from the calculation of correct mean response times (RT) and analysed separately. RT that was ±3SDs from the mean per participant and per condition (1.4%) were removed from the correct RT data and not analysed further. Cell means for correct RT in target-present and target-absent trials for Experiment 2 are shown in Fig. 4. A summary of the findings from the analyses carried out is presented in Table 4.

**Table 4:** Experiment 2: Summary of findings in Experiments 2a (neutral distractors) and 2b (appealing distractors)

	Experiment 2a – Neutral distractors				Experiment 2b – Appealing distractors			
Target present	*df*	*F*	*p*	*ηp*^*2*^	*df*	*F*	*p*	*ηp*^*2*^
RT analyses								
Set size	3,75	70.31	<.001	.74	3,72	80.94	<.001	.77
Complexity	1,25	41.64	<.001	.62	1,24	79.22	<.001	.77
Appeal	1,25	20.77	<.001	.45	1,24	0.47	.50	-
Complexity × Appeal	1,25	5.70	.02	.19	1,24	8.27	<.01	.26
Set Size × Complexity	3,75	0.76	.52	.50	3,72	3.71	<.01	.13
Set Size × Appeal	3,75	1.14	.34	-	3,72	0.59	.62	-
Set Size × Complexity × Appeal	3,75	0.88	.45	-	3,72	1.29	.27	-
Slopes analyses								
Complexity	1,25	1.27	.27	-	1,24	8.73	<.007	.41
Appeal	1,25	1.62	.21	-	1,24	0.70	-	-
Complexity × Appeal	2,25	0.42	.52	-	2,24	0.68	-	-
Target absent	*df*	*F*	*p*	*ηp*^*2*^	*df*	*F*	*p*	*ηp*^*2*^
RT analyses								
Set size	3,75	148.99	<.001	.86	3,72	105.17	<.001	.81
Complexity	1,25	30.84	<.001	.55	1,24	59.66	<.001	.71
Appeal	1,25	9.63	.005	.28	1,24	3.05	.09	-
Complexity × Appeal	1,25	19.13	<.001	.43	1,24	4.08	.05	.14
Set Size × Complexity	3,75	4.34	.007	.15	3,72	17.75	<.001	.42
Set Size × Appeal	3,75	0.75	.52	-	3,72	1.93	.13	-
Set Size × Complexity × Appeal	3,75	1.14	.34	-	3,72	0.39	.76	-
Slopes analyses								
Complexity	1,25	10.68	.003	.30	1,24	40.31	<.001	.63
Appeal	1,25	.69	.41	-	1,24	0.05	.82	-
Complexity × Appeal	2,25	1.81	.19	-	2,24	0.09	.77	-

#### Target-present RT analyses

A repeated-measures ANOVA on *target-present* trials only revealed a significant main effect of Set Size with increasing RT with larger set sizes. The main effect of Complexity was significant with simple icons being found faster than complex icons. The main effect of Appeal was also significant with appealing icons found significantly faster than unappealing icons. The Complexity × Appeal interaction was significant. Pairwise comparisons to examine the Complexity × Appeal interaction showed longer search RT for complex unappealing compared to complex appealing targets (difference = 54 ms, *t*(25) = 4.69, *p* < .001, *d* = .92), but a nonsignificant difference between simple appealing and unappealing targets (difference = 15 ms, *t*(25) = 1.46, *p* = .16, *d* = .29). There were no other significant interactions.

#### Target-present slopes analyses

Search slopes for *target-present* trials were submitted to a 2 (Complexity: complex vs. simple) × (Appeal: appealing vs. unappealing) repeated-measures ANOVA. Neither the main effect of Appeal, nor Complexity were significant, and neither was their interaction.

Overall, both *visual complexity* and *appeal* influenced *search RT* when the target was present in the array, with faster searches when the target was simple and when the targets were appealing. *Search efficiency* was influenced neither by visual complexity nor by appeal.

#### Target-absent RT analyses

A repeated-measures ANOVA for *target-absent* RT showed a significant main effect of Set Size with increasing RT as the number of distractors increased. The main effect of Complexity was significant as was the main effect of Appeal. The Complexity × Appeal interaction was significant. Pairwise comparisons to examine the interaction, showed that target-absent RT was overall faster when looking for complex appealing targets, compared to unappealing targets (difference = 65 ms, *t*(25) = 5.67, *p* < .001, *d = 1.11*) and while there was no difference in search RT between simple appealing and unappealing target (difference = 17 ms, *t*(25) = 1.38, *p* = .18, *d =.27*). The only other significant interaction was between Set Size and Complexity, reflecting steeper RT slopes for complex compared to simple targets (see slopes analyses below). Neither the Set Size × Appeal nor the three-way interaction were significant.

#### Target-absent slopes analyses

Search slopes for *target-absent* trials were submitted to a 2 (Complexity: complex vs. simple) × (Appeal: appealing vs. unappealing) ANOVA, showed a significant main effect of Complexity, with steeper slopes for complex (60 ms/item) compared to simple (50 ms/item) target icons. Neither the main effect of Appeal nor the interaction were significant.

Overall, Experiment 2a results largely replicated the pattern of results in Experiment 1 when appeal was defined independently (based on previous ratings). For target-absent trials *appeal* interacted with *visual complexity* to influence overall *search RT*: search was terminated later for unappealing compared to appealing targets, with this difference being larger for complex compared to simple target icons. *Search efficiency* was determined only by *visual complexity*, with steeper search slopes when searching for complex compared to simple targets.

## Experiment 2b: Appealing distractors

In Experiment 2b appealing and unappealing targets appeared among appealing distractors (see Table 4). All other aspects of the methodology were identical to Experiment 1 and 2a.

### Method

#### Participants

Twenty-six new participants, 17 females and nine males, with ages ranging from 19 to 55 years (*M =* 25.09, *SD* = 10.45) took part in Experiment 2b. Participants were recruited via an advertisement displayed on televisions around Swansea University Singleton Campus Psychology students received participant pool credits, while non-psychology participants received £6 for their time. Participants were all native English speakers and reported normal or corrected-to-normal vision. The study was approved by the College of Human & Health Sciences, Swansea University, Research Ethics Committee.

#### Apparatus and materials

The same apparatus was used as in Experiment 1 and 2a. Target icons were the same as those used in Experiment 2a – varying orthogonally in terms of appeal and visual complexity while matched in terms of concreteness and familiarity. However, a new set of 72 distractor icons was used in Experiment 2b, with the criterion that they were all rated high in terms of aesthetic appeal (greater than a rating of 3). The characteristics of the distractor icons used in Experiment 2b appear in Table 1, along with the comparisons between target and distractor icons on each icon characteristic.

#### Design, procedure and analyses

The design and procedure for Experiment 2b was identical to that of Experiment 2a, with the exception of the level of appeal of the distractor icons. Unlike Experiment 2a, only appealing icons were chosen as distractors, based on the appeal ratings from McDougall and Reppa (2008).

### Results

Trials with incorrect responses (4.42%) and correct trials with responses that were ±3 SDs from the mean per participant per condition (1.6%) were removed from the data and were not analysed further. One participant’s data was removed from the calculation of mean RT as their overall RT exceeded 3 standard deviations from the group mean. Mean correct response times (ms) for the remaining 25 participants per condition are shown in Fig. 4. The findings from the analyses carried out appears in Table 4.

#### Target-present RT analyses

A repeated-measures ANOVA on correct RT of *target-present* trials only, revealed a significant main effect of Set Size and a significant main effect of Complexity. The main effect of Appeal was no longer significant but there was a significant Complexity × Appeal interaction. Pairwise comparisons showed no difference in RT between *simple* appealing and unappealing target icons, *t*(24) = 1.82, *p* = .08, but search times were faster for *complex* appealing compared to *complex* unappealing icons, *t*(24) = 2.51, *p* < .01, *d* = .50. The interaction between Complexity × Set Size was also significant with steeper search slopes for complex compared to simple target icons (see slopes analyses below). There were no other significant interactions, including the theoretically relevant Set Size by Appeal interaction.

#### Target-present slopes analyses

Search slopes for *target-present* trials were submitted to a 2 (Complexity: complex vs. simple) × (Appeal: appealing vs. unappealing) repeated-measures ANOVA. The main effect of Complexity was significant with steeper slopes for complex (28 ms/item) compared to simple targets (19 ms/item). Neither the main effect of Appeal nor the interaction were significant.

#### Target-absent RT analyses

A repeated-measures ANOVA on correct RT of *target-absent* trials, showed a significant main effect of Set Size significant main effect of Complexity as well as a significant Set Size × Complexity interaction reflecting steeper search slopes for complex targets (see slopes analyses below). The main effect of Appeal was not significant. The Complexity × Appeal interaction was significant, with faster search termination RTs for complex appealing targets compared to unappealing counterparts (*t*(24) = 2.57, *p* = .001, *d* = .51), but no difference in search RT between simple appealing and unappealing targets [*t*(24) = 0.13, *p* = .90). None of the other interactions were significant.

#### Target-absent slopes analyses

Search slopes for *target-absent* trials were submitted to a 2 (Complexity: complex vs. simple) × (Appeal: appealing vs. unappealing) ANOVA. Only the main effect of Complexity was significant with steeper slopes for complex (80 ms/item) compared to simple targets (63 ms/item). Neither the main effect of Appeal, nor the interaction were significant.

In summary, when distractor icons were appealing, *search RT* and *search efficiency* for target-present and target-absent trials were only influenced by the *visual complexity* of the target icon. Target appeal no longer facilitated search or search termination times.

### Comparison between Experiment 2a and Experiment 2b

Experiments 2a and 2b were identical in every aspect apart from the level of appeal of the distractor icons. The aim was to examine the effect that distractor appeal might have on search efficiency, as captured in search slopes, for appealing and unappealing target icons. A summary of the results from the analyses comparing the search slopes in Experiments 2a and 2b appears in Table 5.

**Table 5:** Experiment 2: Summary of analyses comparing search slopes in Experiments 2a and 2b

	*df*	*F*	*p*	*ηp*^*2*^
Target-present search slopes				
Target Complexity	1,49	8.96	.004	.16
Target Appeal	1,49	0.01	.97	-
Distractor Appeal	1,49	4.19	.05	.08
Target Complexity × Target Appeal	1,49	0.76	.39	-
Target Complexity × Distractor Appeal	1,49	2.19	.14	-
Target Appeal × Distractor Appeal	1,49	4.58	.04	.09
Target Complexity × Target App × Distractor App	1,49	0.01	.92	-
Target-absent search slopes				
Target Complexity	1,49	40.99	<.001	.45
Target Appeal	1,49	0.45	.51	-
Distractor Appeal	1,49	4.51	.04	.07
Target Complexity × Target Appeal	1,49	1.17	.28	-
Target Complexity × Distractor Appeal	1,49	2.62	.11	-
Target Appeal × Distractor Appeal	1,49	0.32	.57	-
Target Complexity × Target App × Distractor App	1,49	0.98	.33	-

#### Comparison of target-present slopes

A mixed 2 (Complexity: complex vs. simple) × 2 (Target Appeal: appealing vs. unappealing) × 2 (Distractor Appeal: neutral vs. appealing) ANOVA was carried out on search slopes for target-present trials, with Distractor type as the between-participants variable. There was a significant main effect of Complexity steeper slopes for complex compared to simple targets, and of Distractor Appeal with steeper slopes when distractors were appealing compared to when they were of medium appeal. Target appeal interacted with Complexity, with *steeper slopes for complex unappealing compared to complex appealing targets*, *t*(49) = 2.02, *p* = .04, *d* = .28, but no such significant difference between simple appealing and unappealing targets, *t*(49) = .52, *p* = .60. Target Appeal did not have a significant main effect on search slopes but it interacted with Distractor Appeal. Pairwise comparisons confirmed that *appealing targets yielded flatter slopes when distractors were neutral in appeal*, (17 ms/item) compared to when *distractors were appealing* (25 ms/item), *t*(49) = 3.07, *p* = .003, *d* = .86. Unappealing targets yielded similar slopes irrespective of whether they appeared among neutral or appealing distractors (20 ms/item and 22 ms/item, respectively), *t*(49) = .41, *p* = .68, *d* = .11. The Distractor Appeal × Complexity interaction was not significant and neither was the three-way interaction.

In summary, when the target was present, distractor appeal influenced search slopes overall, with more inefficient searches when distractors were of high compared to neutral appeal. Distractor appeal also interacted with target complexity to yield steeper slopes for complex target icons when they were surrounded by appealing compared neutral appeal distractors. Critically, distractor appeal interacted with target appeal, with steeper slopes for appealing targets amongst appealing distractors compared to neutral appeal distractors.

#### Comparison of target-absent slopes

The same ANOVA on *target-absent* trials, showed a significant main effect of Distractor Appeal with steeper search slopes when search was among appealing distractors (71 ms/item) compared to neutral distractors (55 ms/item). Target Complexity was also significant on target-absent slopes with steeper slopes for complex (70 ms/item) compared to simple targets (56 ms/item). There were no significant interactions.

### Discussion

Experiment 2 examined the effect of distractor aesthetic appeal on search times for appealing and unappealing targets. Distractor appeal influenced search slopes, slowing search times down overall. Distractor appeal also interacted with target appeal: searching for appealing targets among appealing distractors resulting in more inefficient search compared to searching for appealing targets among distractors of medium, or neutral, levels of appeal. Meanwhile, unappealing targets did not benefit from being surrounded by appealing distractors. In other words, lack of appeal did not lead to more efficient searches when looking through appealing distracting stimuli, compared to when looking through icons of neutral appeal.

Taken together the findings from Experiments 2a and 2b suggest that what leads to faster searches for appealing targets is the attribute of appeal itself, as opposed to target-distractor similarity. Previous literature shows that the speed at which distractors are rejected can depend on target-distractor similarity (e.g., Duncan & Humphreys, 1989) in terms of low-level features but also in terms of non-perceptual attributes. Here, the target-distractor similarity was based on appeal – a high-level and subjective attribute of objects. However, as unappealing target icons were defined by the absence of appeal (e.g., Wolfe, 2001; Wolfe & Horowitz, 2004) and absence is not a feature that can influence search slopes (e.g., Treisman, 1986), the consistently steep search slopes for unappealing targets regardless of distractor appeal, suggest that they lacked the attribute of appeal that was needed to facilitate search times.

Search slopes were steeper when distractors were appealing, compared to when they were neutral in appeal, and that was the case both for target-present and target-absent trials. This suggests that appealing ‘crowds’ are searched slower than crowds of neutral appeal. The slower search among appealing distractors, may be due to the difficulty in disengaging from appealing distractors (e.g., Fox et al., 2002), or because appeal constricts the focus of attention leading to steeper search slopes (e.g., Fenske & Eastwood, 2003). Therefore, despite the lack of evidence for pre-attentive processing of appeal (as would be evidenced by flatter search slopes) or search inequality between appealing and unappealing targets, the steeper search slopes among appealing distractors, suggest that the appeal of items in a search array influences search performance.

## Experiment 3: The effect of target concreteness and target appeal on visual search

This is the first examination, to our knowledge, of whether concreteness – the degree to which an image refers to something in the real world – which is a key characteristic of pictures, icons and symbols, is an attention guiding attribute. Previously icon concreteness has been associated with better performance in localisation and identification tasks (e.g., Green & Barnard, 1990; Isherwood et al., 2007; McDougall & Isherwood, 2009; Rogers & Oborne, 1987; Stotts, 1998). This makes it a good candidate as a possible icon characteristic which may improve search efficiency as well as search speeds. This experiment mirrors Experiment 1 in terms of design, procedure and analyses with the primary difference between that target concreteness, rather than target complexity, was varied orthogonally with target appeal.

### Method

#### Participants

Twenty new undergraduate Swansea University students (13 females, seven males), with ages ranging from 19 to 39 years (*M =* 24.12, *SD* = 9.46) and with normal or corrected-to-normal vision, took part in Experiment 3 in exchange for psychology participant pool credits. Participants were naïve to the purpose of the experiment. The study was approved by the College of Human & Health Sciences, Swansea University, Research Ethics Committee.

#### Apparatus and materials

The apparatus was the same as in the previous experiments. Forty target icons and seventy-two distractor icons were chosen from the icon corpus in McDougall et al. (1999). The target icons orthogonally varied their rated Concreteness and Appeal, leading to four icon types: appealing concrete, appealing abstract, unappealing concrete, and unappealing abstract (Table 6). Ratings of visual complexity, concreteness, and familiarity were obtained from McDougall et al. (1999), and ratings of appeal were obtained by McDougall and Reppa (2008). A set of univariate ANOVAs showed differences between the Icon Types in terms of rated Concreteness, Familiarity, and Appeal, and the lack of difference in terms of Visual Complexity (see Table 6 for details). Seventy-two icons from the original icon corpus were used as distractors, whose characteristics (visual complexity, concreteness, familiarity, and appeal) appear in Table 1. Distractors were chosen to be neutral in terms of rated appeal. Comparisons between target and distractors icons in Experiment 3 appear in Table 6.

**Table 6:** Experiment 3: Mean ratings (and standard deviations) for icon concreteness, aesthetic appeal, visual complexity and familiarity for each type of icon, and the results of one-way analyses and Newman-Keuls comparisons examining differences between icon ratings in each condition in Experiment 3

Icon characteristics	Target icon types				(a) Comparisons between target icon types		Neutral Distractors **ND**	(b) Comparisons between target icons and distractors	
	Appealing Abstract **AA**	Appealing Concrete **AC**	Unappealing Abstract **UA**	Unappealing Concrete **UC**	F-value	Newman-Keuls comparisons		F-value	Newman-Keuls comparisons
Appeal	3.52 (0.18)	3.5 (0.10)	2.51 (0.11)	2.58 (0.17)	*F* (3 36)=204.89, *p*<.001	AA=AC>UA=UC	3.00 (0.40)	*F* (4, 107)=22.07, *p*<.001	AA=AC>ND UA=UC<ND
Complexity	3.00 (0.53)	2.67 (0.95)	3.1 (0.75)	3.22 (0.76)	*F* (3, 36)=0.96, *p*=.42	AA=AC=UA=UC	2.55 (0.77)	*F* (4, 107)=2.29, *p*=.03	AA=AC=ND UA=UC=ND
Concreteness	2.06 (0.35)	4.57 (0.25)	2.17 (0.20)	4.55 (0.15)	*F* (3,36)=319.71, *p*<.001	AA=UA<AC=UC	3.18 (0.98)	*F* (4, 107)=21.95, *p*<.001	AA=UA<ND AC=UC>ND
Familiarity	2.47 (0.96)	3.7 (0.43)	2.08 (0.57)	3.78 (0.35)	*F* (3, 36)=18.89, *p*<.001	AA=UA<AC=UC	2.86 (0.90)	*F* (4, 107)=8.36, *p*<.001	AA=UA<ND AC=UC>ND

*Note.* The Appeal values and statistics are from McDougall and Reppa (2008), and the Complexity, Concreteness, and Familiarity values are from McDougall et al. (1999)

As shown in Table 6 the four icon types differed not only in terms of Concreteness but also in terms of Familiarity. Concreteness and familiarity are highly correlated and their effects cannot easily be separated (see McDougall et al., 1999, and Prada et al., 2016). We carried out two sets of analyses – in the first we coded the icons in terms of their independently rated concreteness and appeal, and in the second in terms of their rated familiarity and appeal. When the icons were coded in terms of Familiarity and Appeal, there were 13 icons for familiar appealing, ten icons for familiar unappealing, seven icons for unfamiliar appealing, and ten icons for unfamiliar unappealing.

#### Design and procedure

A 2 (Target Presence: present vs. absent) × 4 (Set Size: 3, 6, 9, or 12) × 2 (Concreteness: concrete vs. abstract) × 2 (Appeal: appealing vs. non-appealing) repeated-measures design was used, yielding 32 within-participant conditions. There were ten trials per condition yielding a total of 320 trials per participant. The dependent measure was RT.

The procedure of the visual search task was identical to that in Experiment 1.

### Results

**Fig. 5:**
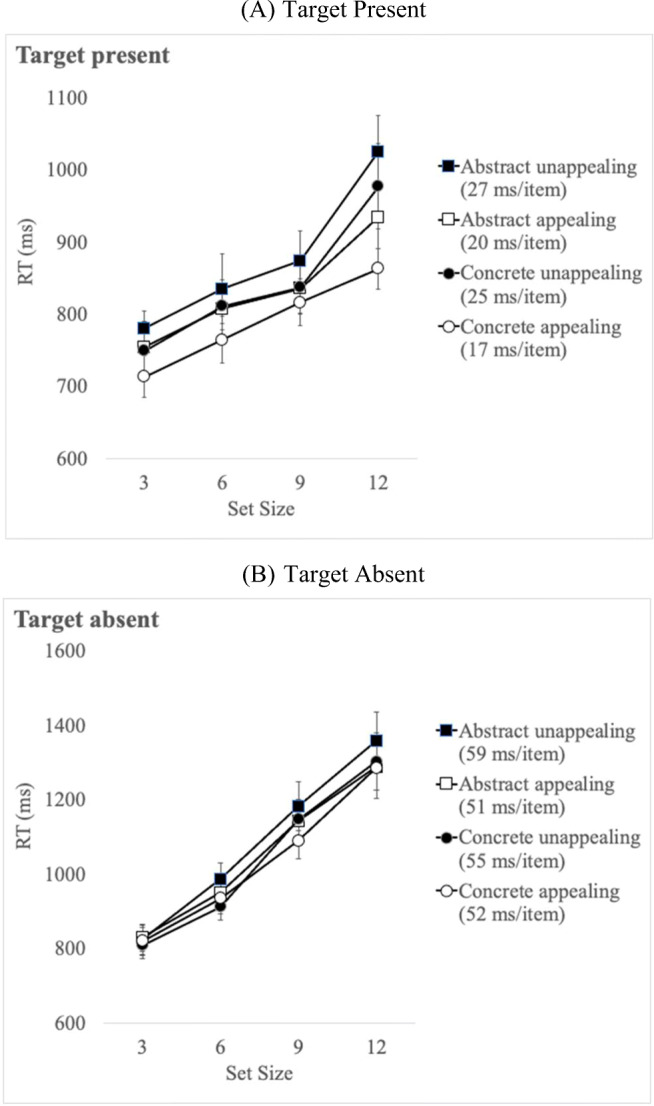
Mean correct response time (RT) per condition for (**a**) target-present trials and (**b**) target-absent trials in Experiment 3 where target icons differed in *appeal and concreteness* and appeared among neutral appeal distractors. Error bars indicate standard error of the mean

Error rates were low accounting for about 6.2% of all trials (4.6% misses and 1.6 % false alarms). Error trials were removed from the correct RT analyses, prior to estimating the mean RT per condition. Correct trials with responses that were ±3 SDs from the mean per participant per condition (0.33%) were excluded from the analyses. Correct mean RT per condition for target-present and target-absent trials appear in Fig. 5. The summary of results from the analyses of RT and slope data of Experiment 3 appears in Table 7.

**Table 7:** Experiment 3 summary of analyses

Target present	*df*	*F*	*p*	*ηp*^*2*^
RT analyses				
Set size	3,60	41.08	<.001	.67
Concreteness	3,60	18.55	<.001	.48
Appeal	3,60	18.60	<.001	.48
Concreteness × Appeal	1,20	0.17	.68	-
Set size × Concreteness	3,60	0.68	.56	-
Set size × Appeal	3,60	0.12	.94	-
Set size × Concreteness × Appeal	3,60	1.66	.18	-
Slopes analyses				
Concreteness	1,20	0.36	.55	-
Appeal	1,20	0.01	.92	-
Concreteness × Appeal	1,20	0.51	.48	-
Target absent	*df*	*F*	*p*	*ηp*^*2*^
RT analyses				
Set size	3,60	165.89	<.001	.89
Concreteness	3,60	11.14	.003	.36
Appeal	3,60	5.82	.02	.22
Concreteness × Appeal	1,20	2.96	.28	-
Set size × Concreteness	3,60	1.96	.13	-
Set size × Appeal	3,60	1.23	.28	-
Set size × Concreteness × Appeal	3,60	2.31	.08	-
Slopes analyses				
Concreteness	1,20	0.93	.35	-
Appeal	1,20	3.48	.08	-
Concreteness × Appeal	1,20	0.45	.51	-

#### Target-present RT analyses

A 4 (Set Size: 3, 6, 9, 12) × 2 (Concreteness: concrete vs. abstract) × 2 (Appeal: appealing vs. non-appealing) repeated-measures ANOVA was carried out on correct RT of *target-present* trials. The main effect of Set Size was significant with RT increasing with larger set sizes. The main effect of Concreteness was significant faster search termination times for concrete icons compared to abstract icons. The main effect of Appeal was also significant with faster search termination times for appealing icons compared to unappealing icons. None of the interactions were significant.

#### Target-present slopes analyses

A 2 (Concreteness: concrete vs. abstract) × 2 (Appeal: appealing vs. unappealing) repeated-measures ANOVA on search slopes, showed no significant main effect of Concreteness no significant main effect of Appeal and no significant interaction.

In summary, when the target was present, *appeal* and *concreteness* independently influenced *search RT* - it took less time to find the target if it was appealing than unappealing, and when it was concrete, as opposed to abstract. Neither appeal nor concreteness however influenced *search efficiency* when the target was present.

#### Target-absent RT analyses

A 4 (Set Size: 3, 6, 9, 12) × 2 (Concreteness: concrete vs. abstract) × 2 (Appeal: appealing vs. non-appealing) repeated-measures ANOVA was carried out on correct RT of *target-absent* trials. This showed a significant main effect of Set Size with RT increasing with larger set sizes. The main effect of Concreteness was significant with faster termination times for concrete compared to abstract target icons. The main effect of Appeal was also significant with faster termination times when looking for appealing compared to unappealing targets. There were no significant interactions.

#### Target-absent slopes analyses

A 2 (Concreteness: concrete vs. abstract) × 2 (Appeal: appealing vs. unappealing) repeated-measures ANOVA on search slopes for target-absent trials, showed a significant main effect of Concreteness. There was no significant main effect of Appeal but the interaction was significant simple effects analysis confirmed that slopes were steeper in unappealing compared to appealing target trials when targets were abstract (*p* < .05), but not when they were concrete (*p* > .05).

Overall, when the target was absent from the array, *appeal* and *concreteness* influenced *search RT*, with search terminated earlier when looking for appealing compared to unappealing targets, and when looking for concrete compared to abstract targets. *Search efficiency* was influenced by the *interaction between appeal and concreteness* – search was more efficient when looking for appealing as opposed to unappealing targets, especially when they were abstract.

### The role of icon familiarity

A further set of analyses was carried out, with the icons re-coded in terms of Familiarity instead of Concreteness. This was done in order to systematically contrast and compare the effects of familiarity vs concreteness on visual search. This was because it was difficult to control the effects of familiarity separately from concreteness as a result of their close correlation.

#### Target-present RT analyses

**Fig. 6:**
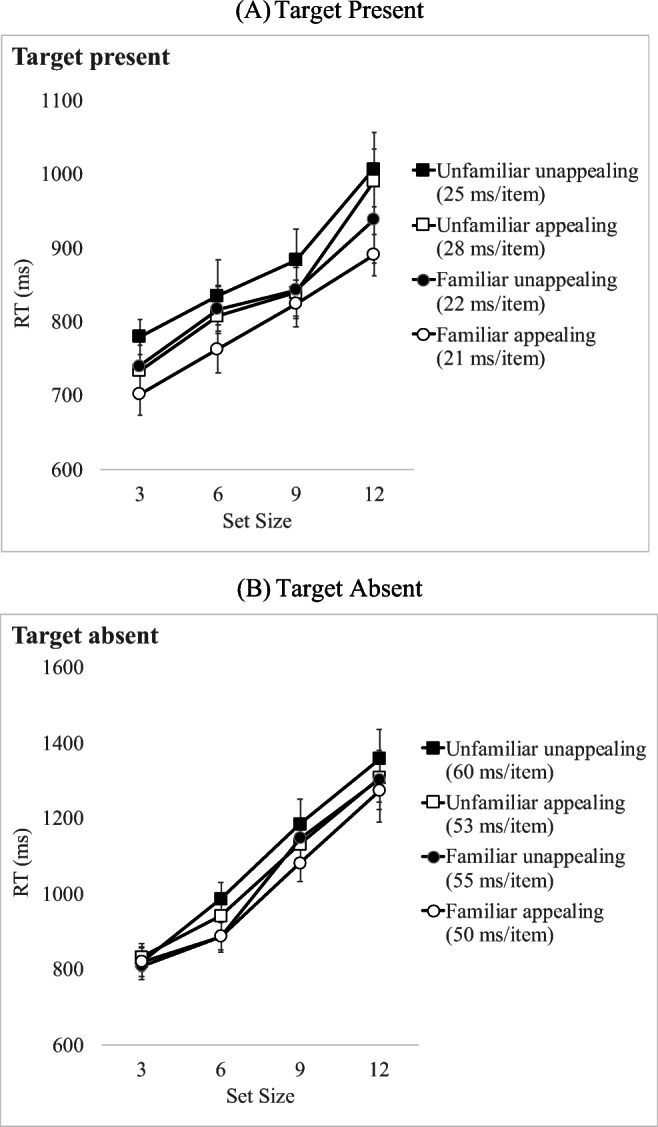
Mean correct response time (RT) per condition for (**a**) target-present trials and (**b**) target-absent trials in Experiment 3 where target icons differed in *appeal and familiarity* and appeared among neutral appeal distractors. Error bars indicate standard error of the mean

Correct cell mean RT appear in Fig. 6, and results of the analyses appear in Table 8. A 2 (Familiarity: familiar vs. unfamiliar) × 2 (Appeal: appealing vs. unappealing) × 4 (Set size: 3, 6, 9 and 12) repeated-measures ANOVA on target-present RT showed that all three main effects were significant. Correct RTs increased with Set Size, familiar icons were found faster than unfamiliar ones and appealing icons were found faster than unappealing ones. There were no significant interactions.

**Table 8:** Experiment 3 summary of analyses based on icon familiarity and appeal (see text for details)

Target present	*df*	*F*	*p*	*ηp*^*2*^
RT analyses				
Set size	3,57	49.20	<.001	.72
Familiarity	1,19	22.43	<.001	.54
Appeal	1,19	4.57	.05	.19
Familiarity × Appeal	1,19	0.20	.66	-
Set size × Familiarity	3,57	1.71	.17	-
Set size × Appeal	3,57	0.05	.98	-
Set size × Familiarity × Appeal	3,57	0.20	.66	-
Slopes analyses				
Familiarity	1,19	1.62	.22	-
Appeal	1,19	0.07	.79	-
Familiarity × Appeal	1,19	0.20	.66	-
Target absent	*df*	*F*	*p*	*ηp*^*2*^
RT analyses				
Set size	3,57	73.81	<.001	.79
Familiarity	1,19	8.66	.008	.31
Appeal	1,19	2.79	.11	-
Familiarity × Appeal	1,19	0.24	.63	-
Set size × Familiarity	3,57	1.10	.36	-
Set size × Appeal	3,57	2.30	.09	-
Set size × Familiarity × Appeal	3,57	0.25	.62	-
Slopes analyses				
Familiarity	1,19	0.75	.40	-
Appeal	1,19	3.77	.07	-
Familiarity × Appeal	1,19	0.09	.76	-

#### Target-present slopes analyses

A 2 (Familiarity: familiar vs. unfamiliar) × 2 (Appeal: appealing vs. unappealing) repeated-measures ANOVA on search slopes, showed no significant main effect of Familiarity, and only a marginally significant effect of Appeal and no significant interaction.

In summary, when the target was present, *appeal* and *familiarity* independently influenced *search RT* – it took less time to find the target if it was appealing than unappealing, and when it was concrete, as opposed to abstract. As was the case with concreteness and appeal, neither appeal nor familiarity however influenced *search efficiency* when the target was present.

#### Target-absent RT analyses

A 4 (Set Size: 3, 6, 9, 12) × 2 (Familiarity: familiar vs. unfamiliar) × 2 (Appeal: appealing vs. non-appealing) repeated-measures ANOVA was carried out on correct RT of *target-absent* trials. This showed a significant main effect of Set Size with RT increasing with larger set sizes. The main effect of Familiarity was significant with faster termination times for familiar compared to unfamiliar target icons. The main effect of Appeal was not significant, and there were no significant interactions.

#### Target-absent slopes analyses

A 2 (Familiarity: familiar vs. unfamiliar) × 2 (Appeal: appealing vs. unappealing) repeated-measures ANOVA on search slopes for target-absent trials, showed than neither of the two main effects were significant. The interaction was only marginally significant (*p* = .09)

Overall, when the target was absent from the array, *appeal* and *concreteness* influenced *search RT*, with search terminated earlier when looking for appealing compared to unappealing targets, and when looking for concrete compared to abstract targets. *Search efficiency* was not influenced by appeal or familiarity although their interaction approached significance, with a trend for a bigger difference between appealing and unappealing unfamiliar items compared to the difference between appealing and unappealing familiar items.

### Discussion

The current findings showed that search was terminated sooner, and it was faster for concrete compared to abstract icons. This finding confirms that icon concreteness is an important variable in task performance when speed is emphasised for the task (i.e., localisation and search tasks), even when it is not task relevant. However, unlike visual complexity it did not influence search efficiency – search slopes were in the ‘quite inefficient’ range for both abstract and concrete icons and there was no search asymmetry between the two conditions. Therefore, even though concreteness can speed search up it only does so after the item is attended to. Separate analyses showed that the effects of familiarity on visual search are similar to those of concreteness. This is not surprising given their high correlation: concrete icons rely on the use of visual metaphors with the real world, often using familiar objects to represent mean producing a close relationship between the two icon characteristics.

Critically, as in the previous experiments, icon appeal moderated search performance: search was faster when looking for an appealing target, compared to unappealing targets. This finding corroborates previous evidence from a localisation task, showing that appealing icons are localised faster than unappealing icons (e.g., Reppa & McDougall, 2015). However, although previous work found that the beneficial effect of appeal on localisation performance was contingent on task difficulty - i.e., only observed for abstract icons, which are typically harder to localise, we found no such interaction here. Instead, the beneficial effect of appeal was observed in visual search regardless of whether icons were concrete or abstract. This could potentially be due to the difference in task demands. In localisation tasks response times are typically longer (ranging between ~1 s and 1.8 s) and encompass both the time to find the icon in the display and the time to move the cursor to its location. In a visual search task, only time to find the target in the display is measured and that time ranged between ~0.6 and 1.2 s.

As in Experiments 1 and 2, despite the overall effect of appeal on search time, there was no evidence that search was more efficient for appealing targets compared to unappealing targets. Search slopes were generally inefficient at around 20 ms per item. Search for unappealing targets lasted longer for both target-present and target-absent trials. That is, when the target was present unappealing icons took longer to find, and when the target was absent, it took longer to terminate the search for unappealing targets. This pattern of results suggests that unappealing targets may be processed less effectively than appealing targets.

## General discussion

Attractiveness of objects, people, or interfaces is key to much of our everyday life decisions and behaviours, with significant socio-economic impact (e.g., Jylhä & Hamari, 2019). But do attractive objects guide attention to themselves? The overarching aim of the current study was to examine whether aesthetic appeal can influence visual search performance. We manipulated visual complexity and aesthetic appeal of a set of icons, while controlling for other icon characteristics. The key findings were as follows:

(i) Search efficiency was determined by visual complexity, with icons rated as visually simple guiding search more efficiently than those rated as visually complex.
(ii) Visual search performance was *not blind to beauty* – to borrow a term from Gerritsen et al. (2008). Search times for appealing targets were faster than for unappealing targets, across all three experiments.
(iii) There was no evidence that appeal was pre-attentively processed and guided search.
(iv) When *distractors* were appealing, the search advantage for appealing *targets* was reduced, and it was not replaced by advantage for unappealing targets.
(v) Appealing *distractors* took longer than neutral distractors to be rejected - as evidenced by the slower search times and less efficient searches among appealing compared to neutral distractors. Those findings are discussed in turn.

### Visual complexity guides search

The current study is one of the few to date examining the role of visual complexity in a classic visual search task (see also Sun & Firestone, 2021). Previous work using search tasks without manipulating search array size, has found a negative impact of visual complexity icons and symbols on performance (e.g., Byrne, 1993; Gerlach & Marques, 2014; Isherwood et al., 2007; McDougall et al., 2000; McDougall et al., 2006; Scott, 1993; Reppa & McDougall, 2015). Manipulating set size allowed us to examine whether and to what extend visual complexity can influence search efficiency. Search for visually simple targets never approach the ‘efficient range’ of approximately zero ms/item. However, search slopes for simple targets were consistently in the ‘quite efficient’ range (~10 ms/item), while search for complex targets were consistently in the ‘quite inefficient’ range of around 20–30 ms/item. Therefore, although there is no evidence for pre-attentive processing of visual complexity of an image, the current work provides converging evidence from a powerful paradigm that visual simplicity is a powerful determinant of task performance with a variety of stimuli (e.g., websites, icons and signs, and geometric objects).

### Aesthetic appeal does not guide search but influences search times

Although appeal moderated search performance, there was no evidence for pre-attentive detection of appeal in the current studies. The current study shows that aesthetic appeal is another high-level attribute that has been examined for its attention guiding potential and found wanting (see Wolfe & Horowitz, 2017, for a recent review; see, e.g., work on facial emotions – Frischen et al., 2008; Gerritsen et al., 2008; Horstmann, 2007, and self-relevant targets – Wade & Vickery, 2018). In general, higher-level attributes influence task performance but require attention to be processed – the attributes do not, in and of themselves, guide visual search.

That aesthetic appeal may be pre-attentively processed, of course remains a possibility, but it is unlikely for many reasons. First, the pattern of quite inefficient slopes repeated in all three experiments (no slope was less than 10 ms/ item). Second, the procedure used was clearly sensitive to changes in stimuli – the pattern of results changed when we used appealing distractors. This suggests that our participants were sensitive to the appeal of stimuli on the screen. Of course, the absence of evidence for pre-attentive processing does not rule out the possibility that visual appeal is an important object dimension. The aesthetic appeal of a stimulus lies along a continuum, with most stimuli lies somewhere along it. Lack of efficient searches may be due either to distractors never being at zero level of appeal, or to the targets not being sufficiently high in appeal.

### Methodological considerations

The icons used in the current study, and their properties, are published elsewhere (e.g., McDougall et al., 1999) and open to scrutiny and reassessment. It always remains a possibility that another variable might be postulated to underlie the significant effect of appeal on performance shown here. However, the following findings suggest that it is the appeal of icons that drives at least some of the performance changes. First, among appealing distractors only the search performance for appealing targets was impacted compared to when distractors were neutral, while search performance for unappealing targets remained unaffected by the distractors. Second, it is unlikely that there is something specific about the target icons used here that leads to the observed pattern of performance. The same target icons, used in a search and localisation task previously have led to different patterns of performance depending on the induced mood of participants (e.g., Reppa et al., 2021). Furthermore, the same pattern of results regarding the effect of appeal on each times arose when using different target icons (i.e., Experiments 1 and 2 vs. Experiment 3). Therefore, at the current time, aesthetic appeal has proven to have explanatory efficacy above any of the examined dimensions of complexity, concreteness, and familiarity. Although it always remains possible that some unthought of, correlated, stimulus dimension could be affecting performance, any such suggestion needs to first be demonstrated to be plausible within the stimulus set used here.

A key issue relates to the choice of icons as stimuli in the current and previous related studies (e.g., Reppa et al., 2021; Reppa & McDougall, 2015). Researchers interested in aesthetics and its influence on behaviour and performance are likely to wonder what the effect of stimuli with a stronger aesthetic value, and the potential to arouse strong aesthetic emotions might have on experience and behaviour.^1^ There are at least three reasons we do not think the use of icons as aesthetic elucidators is a problem when asking whether aesthetics can influence performance. First, what previously might have been thought to have been the domain of aesthetic appreciation – works of art, music, or even facial attractiveness – has become more generalised with icons and websites being well-recognised. They may be familiar (not least because icons use object metaphors from the real world to convey meaning) and simple (since rapid and effective responding is often required) but this does not mean that they do not elicit an aesthetic response (see McDougall & Reppa, 2008; Prada et al., 2016). Indeed, it could be argued that they are an important stimulus set which is growing and developing rapidly, that is universally used in a way that other stimuli, such as works of art, may not be. As we noted earlier, icon stimuli have increasingly become recognised as important stimuli for study in their own right with respect to appeal because of their frequent use. Furthermore, given that most individuals search for icons on interfaces daily, we would argue that the effect of icon appeal on visual search times is very important indeed and could be vital in interface design where responses are both time- and safety-critical (e.g., cockpit interfaces). We posit that it is high time aesthetic appeal got away from the idea that appeal is a ‘high flown’ concept for the arts and music. It is a basic response to faces (e.g., Becker et al., 2011; Nakamura & Kawabata, 2018), websites, (e.g., Baughan et al., 2020; Lindgaard et al., 2006), and to icons, signs, and symbols (e.g., Reppa et al., 2021; Reppa & McDougall, 2015).

Second, liking responses of the kind that we measure here and experience on a day-to-day basis, can have pervasive influence on our behaviour and, as we show here, on our performance. In recognition of this, there is increasing number of publications of normative ratings for icons and other pictorial stimuli that include ratings of aesthetics. In a recent review of normative studies of pictures, Souza et al. (2020) argued that aesthetic appeal was an important stimulus characteristic and part and parcel of the cognitive processing of both pictures and icons (see also Garrido et al., 2016; McDougall & Reppa, 2008; Prada et al., 2016; Souza et al., 2021). Icon-specific research has also demonstrated the importance of these factors in determining user performance (Reppa et al., 2021; Reppa & McDougall, 2015).

Third, the role of appeal has recently been given increasing prominence in consumer psychology. Research has consistently shown that judgements about webpages are made at an early stage of processing (Lindgaard et al., 2006; Lindgaard et al., 2011) and standardised tools to assess appeal have been developed (e.g., Thielsch & Hirschfeld, 2019). Thus, in both human-computer interaction and consumer psychology rapid processing of appeal and appeal judgements have gained increasing importance although it may differ from original conceptions of aesthetic appreciation as a deliberative conscious process.

### Theoretical considerations

The current findings suggest that appeal might influence performance by enhancing the processing of appealing stimuli. Despite the lack of evidence of appeal guiding attention during search, once the target was attended it was processed faster than unappealing targets. This was especially true when those targets were visually complex (in Exp. 1 independent ratings, and Exps. 2a and 2b), and when set sizes were large (Exps. 1 and 2a). Both findings corroborate previous evidence showing conditional effects of appeal on performance with icons and with websites (e.g., Moshagen et al., 2009; Reppa et al., 2021; Reppa & McDougall, 2015). The conditional effect of appeal on task difficulty is predicted by the affective mediation hypothesis (e.g., Norman, 2004). According to this hypothesis appeal only influences performance under duress, by raising positive affect and thus speeding up processing and response times. The source of the enhanced processing of appealing icons is a topic in need of further investigation. For example, appeal may lead to a target item being examined for longer or at different levels of analysis (e.g., global vs. local analysis) leading to a better perceptual representation of a stimulus. Alternatively, appeal may lead to a better commitment of an item into working memory, leaving more resources to conduct an efficient search for the target^2^. We are currently examining these possibilities.

Obtaining subjective ratings of appeal in Experiment 1 gave us a further insight into the mechanism by which appeal may influence search performance. Specifically, the only time appeal had a ubiquitous effect on performance was when we took into account participants’ own liking ratings: icons that the participants themselves rated highly in appeal were found faster regardless of the icons’ visual complexity. This finding theoretically relevant, as it suggests that *appeal may increase motivation* which in turn can speed up search performance. The current findings lend further support to this notion – we find unconditional effects of appeal on performance for stimuli we personally like and thus find more rewarding (e.g., Della Libera & Chelazzi, 2009; Lee & Shomstein, 2014).

Other work has shown that stimuli that are associated with rewards can become visually salient and accordingly enhance performance in visual cognition tasks (e.g., Hickey et al., 2011; Kiss et al., 2009; Kristjánsson et al., 2010; Lee & Shomstein, 2014). The current finding of steeper search slopes when distractors were appealing compared to neutral are reminiscent of findings from studies examining the effect of reward on visual search tasks, have shown similarly that high-reward distractors can capture attention and thus slow search performance (e.g., Anderson et al., 2011). Overall, the current findings are compatible with the idea that appealing stimuli act like other rewarding stimuli, like food or money, it is harder to disengage from them and they are processed faster than their unappealing counterparts.

### Practical considerations

The current findings are important in the field of HCI because they go beyond the evidence showing that more aesthetically appealing interfaces are *perceived* to be more usable (e.g., Kurosu & Kashimura, 1995; Tractinsky et al., 2000), or those appealing interfaces *become more usable* because users make more of an effort with them (e.g., Wiedenbeck, 1999), they show that appealing icons can be found faster in search arrays, therefore, they *are* more usable. This finding is potentially relevant to different types of stimuli and user experience, where optimising performance could have considerable costs. People can be sensitive to performance costs as small as 150 ms (e.g., Gray & Boehm-Davis, 2000). Such costs can add up during multi-step interactions with interfaces, which can lead to employing strategies to avoid interfaces in favour of those that maximise efficient performance. This is likely to be particularly important for interfaces, such as websites (e.g., de Wulf et al., 2006; Pandir & Knight, 2006; Tarasewich et al., 2001; van Schaik & Ling, 2005), apps (e.g., Jylhä & Hamari, 2019), and mobile phones (e.g., Sauer & Sonderegger, 2011; Sonderegger & Sauer, 2010).

In conclusion, the current findings show that visual aesthetic appeal, as a stimulus characteristic, even when task irrelevant, can lead to efficient performance in visual search tasks. Future work needs to examine the mechanism with which appeal can influence performance, be it via positive affect or some other emotional or cognitive process.

## Footnotes

1 We thank two independent anonymous reviewers for raising this issue.

2 We thank an anonymous reviewer for raising this possibility.
